# Supplementary material for: Sequencing trait-associated mutations to clone wheat rust-resistance gene YrNAM
Source: Nat Commun. 2023 Jul 19;14:4353. doi: 10.1038/s41467-023-39993-2 (PMC10356923; doi:10.1038/s41467-023-39993-2)
Supplement: Supplementary file 1 — Supplementary Information [file 41467_2023_39993_MOESM1_ESM.pdf]

Sequencing trait-associated mutations to clone wheat rust-resistance gene *YrNAM*

Ni *et al.*

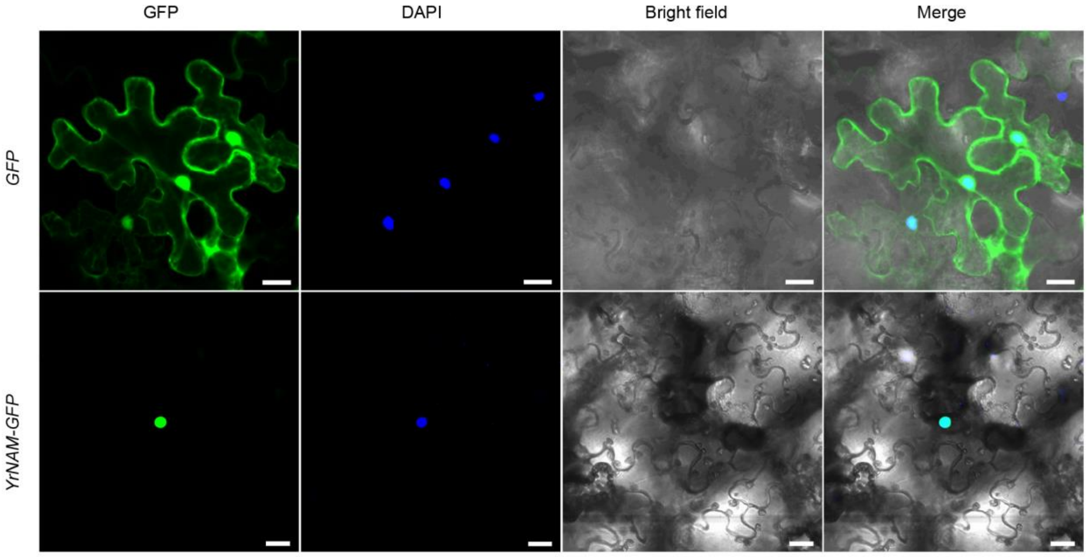

Supplementary Fig. 1. Nuclear localization of YrNAM:GFP.

Subcellular localization of the YrNAM:GFP fusion protein in tobacco leaf cells. GFP expression vector pPZP211-GFP was used as a control. 4′,6-diamidino-2- phenylindole dihydrochloride (DAPI; Sigma, Cat# D9524) was used to stain nuclei. Photographs were taken on a confocal microscope (Zeiss LSM 880 NLO, Germany). Bar = 20 μm. This experiment was performed independently two times with similar results.

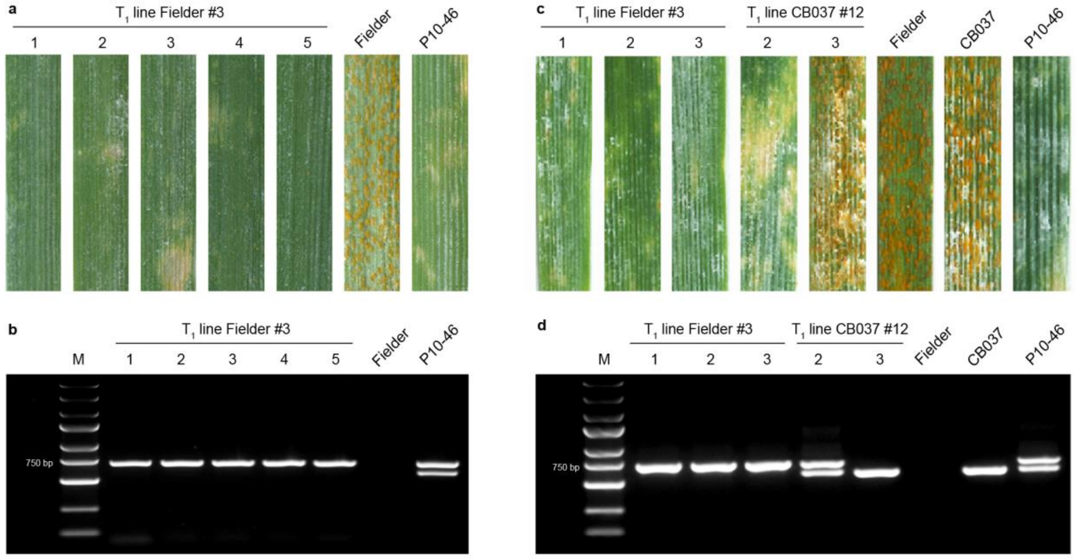

Supplementary Fig. 2. The functional verification of *YrNAM* in stripe rust resistance using additional *Pst* races.

a-b Plants were tested with a mixture of spores of *Pst* race CYR29, CYR31, and CYR32. *YrNAM* line P10-46 and the recipient line Fielder were used as controls. **a** Fielder was highly susceptible (ITs=8-9) while both P10-46 and the five T_1 plants were resistant (ITs=0-2). **b** As revealed by the *YrNAM-F8R7* marker, the five T_1 plants and P10-46 were *YrNAM*-positive and Fielder was *YrNAM*-negative. **c-d** Plants were tested with a natural mixture of *Pst* spores that were collected in Luohe, Henan province of China. The recipient lines Fielder (for Line #3), CB037 (for Line #12), and *YrNAM*-negative transgenic line #12-3 were highly susceptible (ITs=8-9) while P10-46 and the *YrNAM*-positive T_1 plants were resistant (ITs=1-2). The data shown here were performed independently two times with similar results. Source data are provided as a Source Data file.

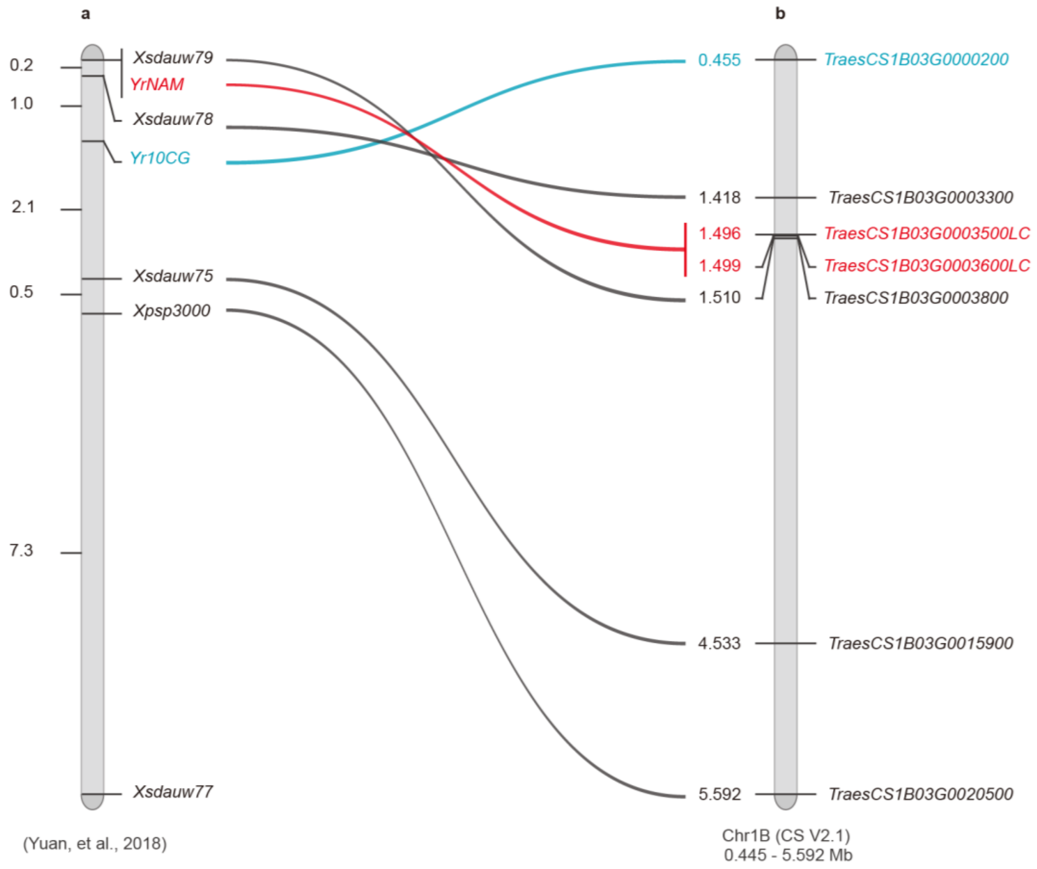

Supplementary Fig. 3. The genetic and physical maps of the *Yr10* region.

a The genetic map was described in Yuan *et al*., 2018. **b** The physical map was drawn based on the Chinese Spring reference genome.

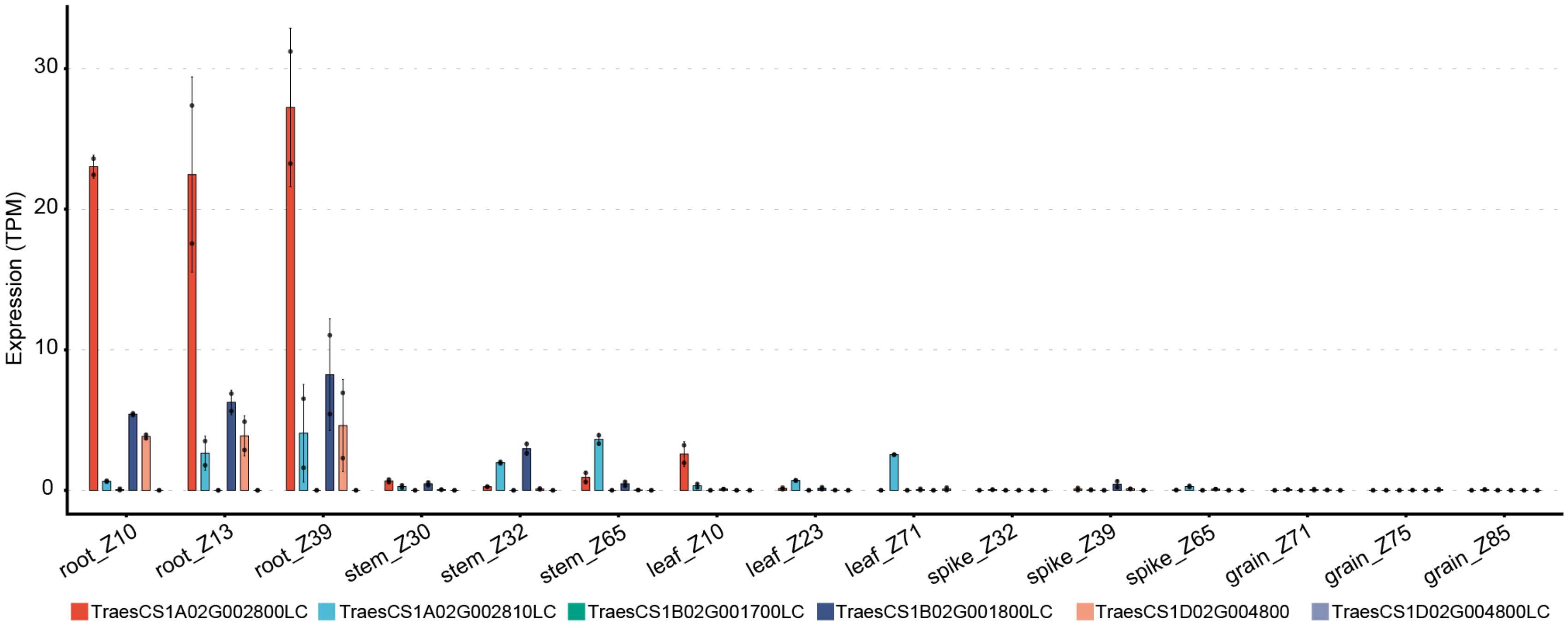

Supplementary Fig. 4. Transcription of six *YrNAM* homologs in cv. Chinese spring.

The RNA-Seq data with two biological replicates were based on IWGSC RefSeq v1.1 (Alaux et al., 2018), and were downloaded from ENA database for accession number ERP004714. The gene expression analysis was performed by using Barplot tool with default parameters in Hiplot Pro (https://hiplot.com.cn/). Each point represents the transcripts per million (TPM) value, each column represents the mean TPM and error bars are the standard deviation (n = 2). Source data are provided as a Source Data file.

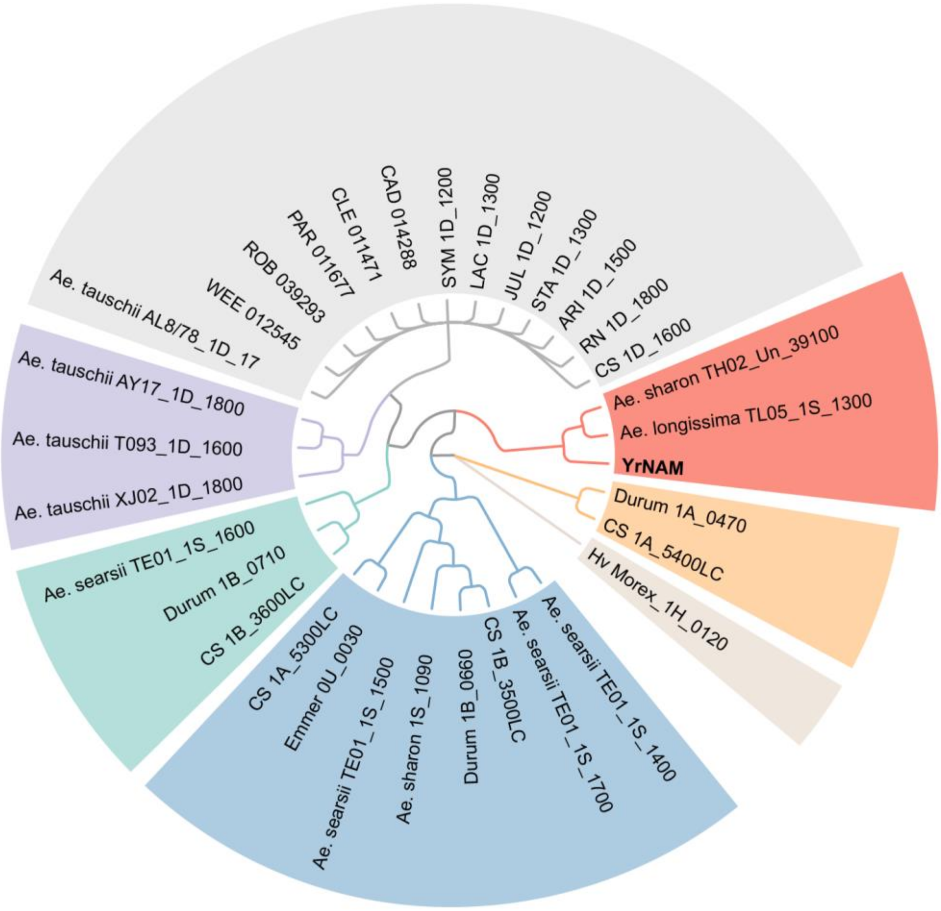

Supplementary Fig. 5. Phylogenetic tree of *YrNAM* homologs.

Blastn searches against the current *Triticeae* data in WheatOmics (Ma et al. 2021) identified thirty-six homologs from hexaploid, tetraploid, and diploid wheats, as well as from four *Aegilops* species. Thirty-two accessions were used to draw the phylogenetic tree. Gene IDs are provided in Supplementary Data 6.

```
                                    10        20        30        40        50
YrNAM                     MEHEEDSFDVENLVCTPTDADLGIPMSPTHLPLPLPLPPSSRHMIMSNTD 50
Ae.longissima_1S_1300     MEHEEDSLDVENLVCTPTDSGL--PMSPAHLHLPLPLPPSSRHMIMNNTD 48
Ae.sharonensis_Un_39100   -------------------------------------------------- 0

                                    60        70        80        90       100
YrNAM                     HVVAESSAQAVAAGKKLEQEFELPADLTCTPTPTPTPTDVELVRDYLRPA 100
Ae.longissima_1S_1300     HVAAQ----AVAAGKKLEQEFELPADLTCTPTST----DVELVRDYLDPA 90
Ae.sharonensis_Un_39100   -------------------------------------------------- 0

                                   110       120       130       140       150
YrNAM                     IQRMQPEERSLWDLINGPAVVNPDLPTMDPCQLPGMIARNSKGGNELRDK 150
Ae.longissima_1S_1300     IQRMQPEERSHWDCIYGPAVVNPDLPTMDPCQLPGMIARNNKGGNELRDK 140
Ae.sharonensis_Un_39100   -----------------------------------MIARNNKGGNELRDK 15

                                   160       170       180       190       200
YrNAM                     YYLTDHNALASFRYFPGLRTTNGYWQTHAYHTAIEAEHNRLVGVKKTMKF 200
Ae.longissima_1S_1300     YYLTDHNALAPFGYSRVLHTTTGFWQTRDY-TVIRAEHNRIVGLKKTMKF 189
Ae.sharonensis_Un_39100   YYLTDHNALAPFGYSRVLHTTNGFWQTHDS-TVIRAEHNRIVGLKKTMKF 64

                                   210       220       230       240       250
YrNAM                     HTYTGAKTNWIMNVYYSLVEGDFFIQEGLVLCYVFETDSAPDYDDNPKCL 250
Ae.longissima_1S_1300     HQHTGAKANWIMNIYYSCKEGDSFLREGLVLCHVFETDSAPDYGDNPKCL 239
Ae.sharonensis_Un_39100   HLHTGAKANWIMNIYYSYKEGDSFLREGLVLCHVFETDSAPDYGDNPKCL 114

                                   260       270       280       290       300
YrNAM                     GCHQGSCSGYHSGQAFAAPS-NASFNSTYPGSRPMHHHDTERRSDPRLSS 299
Ae.longissima_1S_1300     GCHQGACSGHPHDQAFGAPSEDTSF-------KPMHYCPN-RHSDPRLKS 281
Ae.sharonensis_Un_39100   GCHRGACSGHPHDQAFGAPSEDTSF-------KPMHYYPN-RHSDPRLKS 156

                                   310       320       330       340       350
YrNAM                     FMADLEKCLLGDTDDPDNSTDTGNSSARRGDPPTADHGQSKKRKKISDVW 349
Ae.longissima_1S_1300     YMEQLGDLLFSDAD-PDNSADTGNSGAGRGDPPTADHGQCKKRKKISDVW 330
Ae.sharonensis_Un_39100   YMEQLGDLLFSDAD-PDNSADTGNCGAGRGNPPMADHGQSKKRKKISDVW 205

                                   360       370       380       390       400
YrNAM                     DYFTKIFARDINGKVMAYAACNHCCKILSASSKNGTSQLARHACPCKFKP 399
Ae.longissima_1S_1300     DYFTKIFARDINGKVMTYAACNHCCKILSASSKNGTSQLARHACPCKFKP 380
Ae.sharonensis_Un_39100   DYFTKIFARDINGKVVTYAACNHCCKILSASSKNGTSQLARHACPCKFKP 255


YrNAM                     VEAGRNAKD                                          408
Ae.longissima_1S_1300     VEAGRNAKD                                          389
Ae.sharonensis_Un_39100   VEAGRNAKD                                          264
```

Supplementary Fig. 6. Alignments of YrNAM and its homologs in *Aegilops*.

Two *Aegilops* homologs are *Ae. longissima.TL05.1S01G0001300.1* and *Ae. sharonensis.TH02.Un01G0039100.1*, respectively. The sequencing and annotation of *Ae. longissima* (TL05) and *Ae. sharonensis* (TH02) was reported in Li et al. (2022). Alignment was made by MegAlign in DNASTAR Lasergene. Distinct residues are highlighted by red shading.

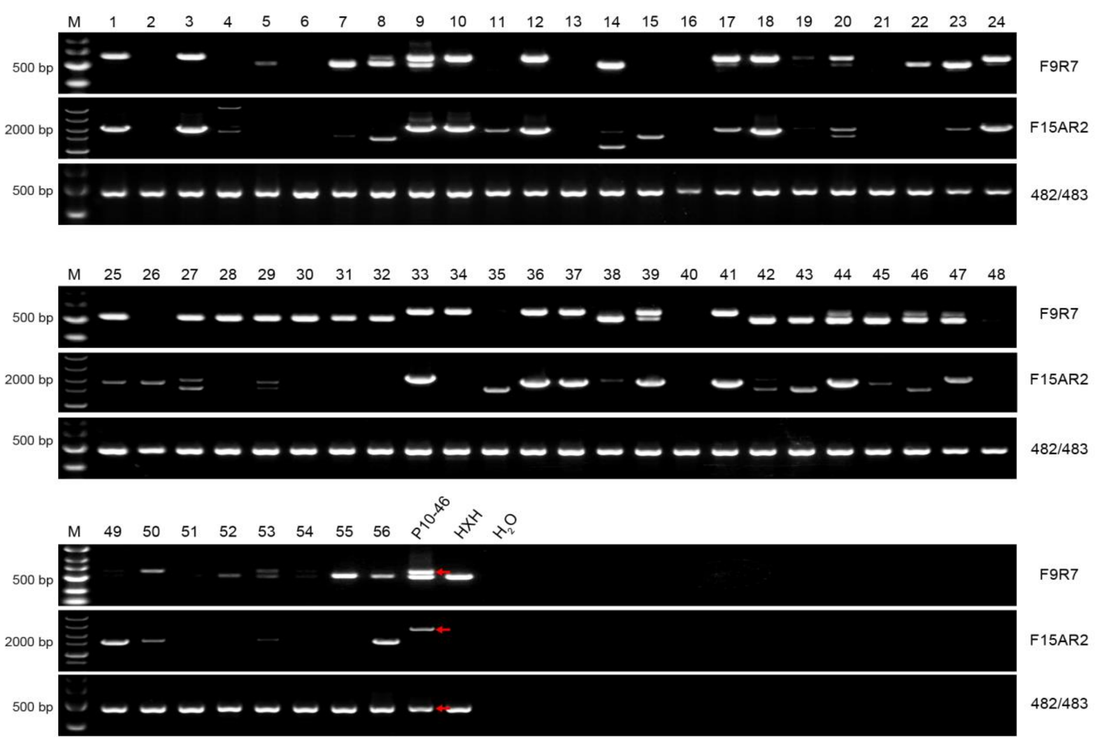

Supplementary Fig. 7. *YrNAM* homologs in *Ae. longissima* and *Ae. sharonensis*. Primer pairs *YrNAM*-F9R7 and F15AR2 were used to amplify *YrNAM* homologs. Primers 482/483 for the actin gene were used to confirm the DNA quality of the samples. Red arrowheads indicate the target bands. Thirteen *Ae. longissima* lines (Lanes 1-13) and 43 *Ae. sharonensis* lines (Lanes 14-56) were analyzed. P10-46 was a positive control. The plant IDs of 56 lines are shown in Supplementary Data 7. This experiment was performed independently two times with similar results. As a note, *YrNAM* has two tandemly duplicated 111 bp repeats located at the 3'-end and involves the stop codon; this duplication also occurs in *TL05.1S01G0001300.1* and *TH02.Un01G0039100.1*, but is absent in other genes or genomic regions in *Triticeae* data in WheatOmics. *YrNAM-F9R7* detects the 111 bp repeats, amplifies a 624 bp fragment covering the unique 111 bp duplications at 3'-end of *YrNAM*, and a non-specific band of 572 bp from the chromosome 1D homolog which does not have *YrNAM*. Using *F9R7*, *YrNAM* homologues were detected in 6 (46%) *Ae. longissima* lines and 16 (37%) *Ae. sharonensis* lines. Using *YrNAM-F15AR2* which amplifies full-length *YrNAM*, approx. two-thirds of *Ae. longissima* and *Ae. sharonensis* lines produced clear bands but of variant sizes. Sequencing of these bands revealed complete open read frames (ORFs) encoding proteins with 75%-79% identities to YrNAM, and revealed that their size variation was due to deletions in introns. These results show a wide distribution and variations of *YrNAM* homologs in *Ae. longissima* and *Ae. sharonensis*. In all five Sitopsis species, *YrNAM* homologs are present in *Ae. longissima*, *Ae. sharonensis*, and *Ae. searsii* (cv. TE01) (Supplementary Fig. 5), but are undetectable in *Ae. bicornis* accession TB01 and *Ae. speltoides* accessions TS01 (Li et al. 2022) and AEG-9674-1 (Avni et al. 2022). Since *Ae. bicornis* and *Ae. speltoides* are species complexes, there may be variation in the presence/absence of *YrNAM* homologs within these species. Source data are provided as a Source Data file.

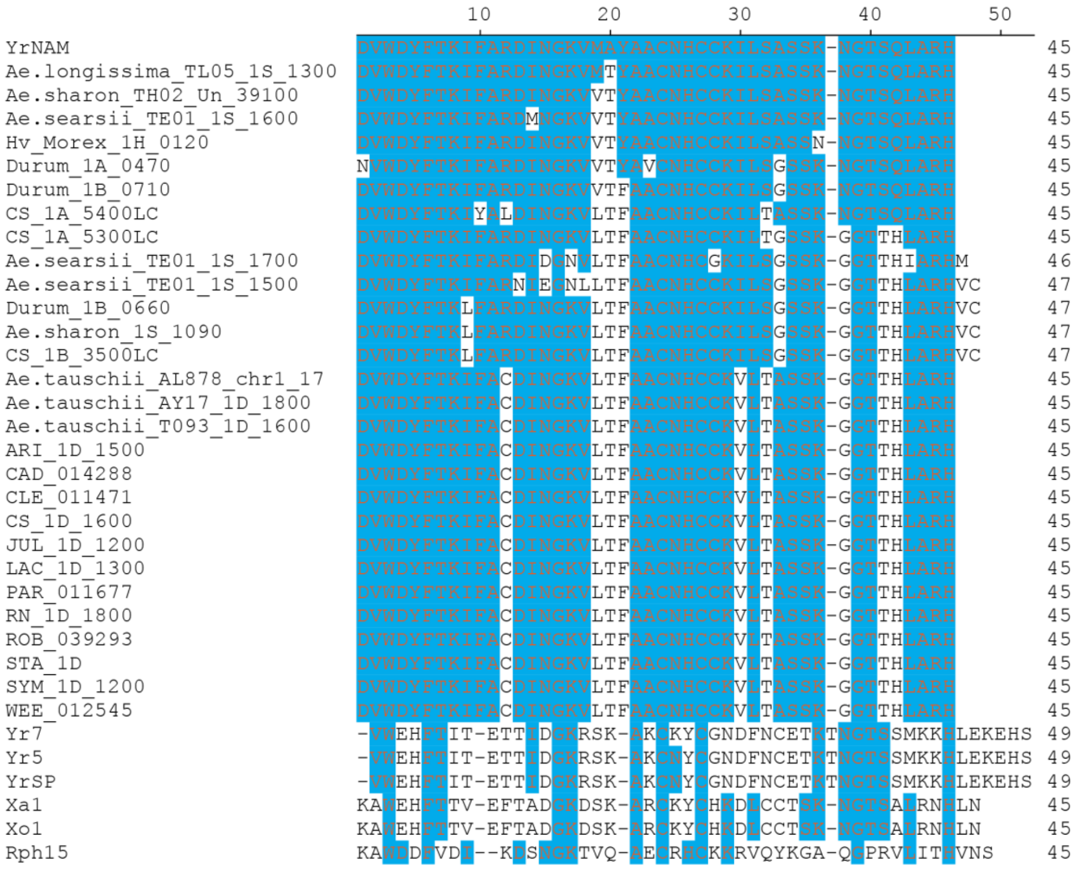

Supplementary Fig. 8. Alignments of ZnF-BED domains from *YrNAM* homologs and known NLR-BED genes.

Using NCBI CD-Search, conserved ZnF-BED domains were extracted from *YrNAM* and 28 homologs, and 6 cloned NLR-BED genes including *Yr7*, *Yr5*/*YrSP*, *Xa1*, *Xo1* and *Rph15*. Gene IDs of the 28 homologs are provided in Supplementary Data 6. Alignment was made by MegAlign in DNASTAR Lasergene. Residues that match those in YrNAM are highlighted by bright turquoise shading.

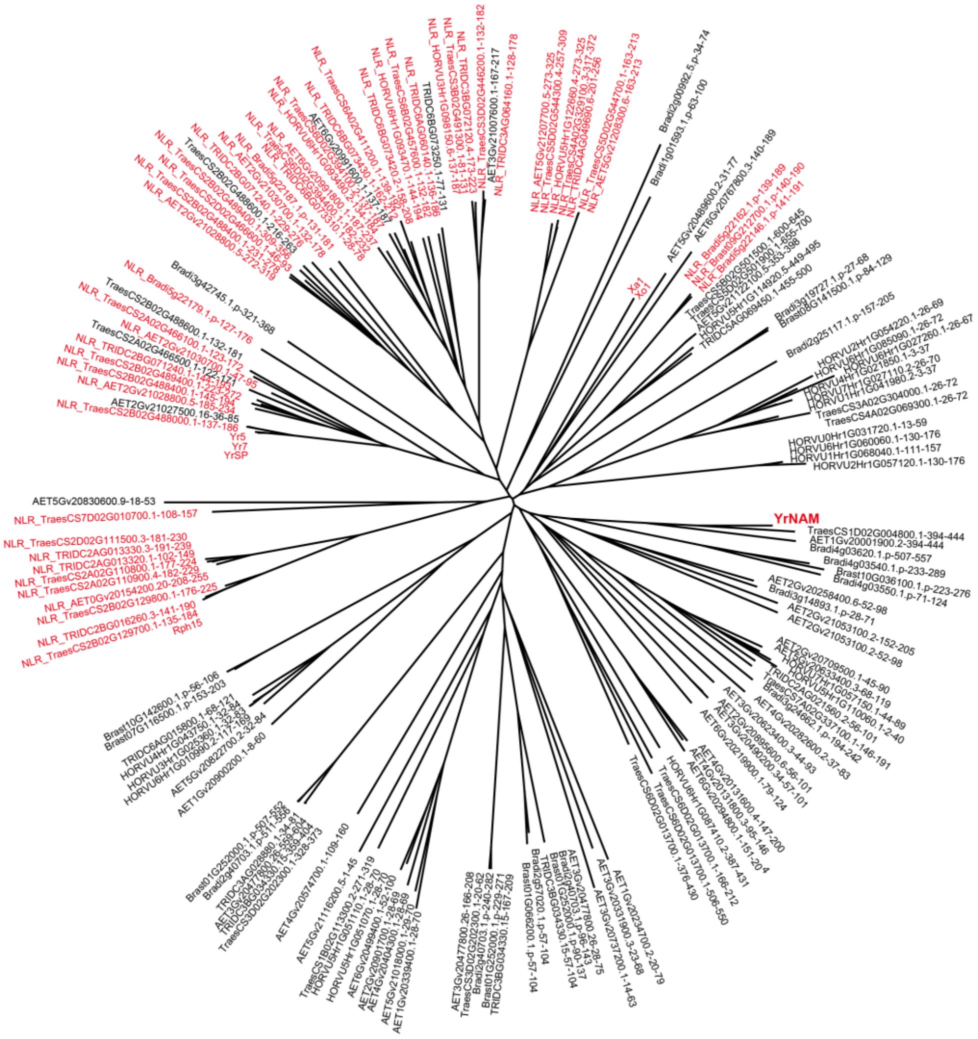

Supplementary Fig. 9. Neighbour-network of ZnF-BED domains from nucleotide-binding leucine-rich-repeat receptors (NLRs) and non-NLRs in the *Poaceae*.

The similarity between ZnF-BED domains of the YrNAM and other BED containing proteins in *Poaceae* was analyzed according to Marchal et al. (2020). Sequences were aligned using MAFFT v7.475 with the L-INS-I method (Katoh and Standley 2013) and the neighbor-network was generated using SplitsTree4 (Huson and Bryant 2006) based on the uncorrected P distance matrix. The dataset includes BED domains from species of *Triticum aestivum* (22 BED-NLR and 15 non-NLRs), *Triticum dicoccoides* (12 BED-NLR and 8 non-NLRs), *Aegilops tauschii* (8 BED-NLR and 36 non-NLRs), *Hordeum vulgare* (4 BED-NLR and 19 non-NLRs), *Brachypodium distachyon* (4 BED-NLR and 14 non-NLRs), *Brachypodium stacei* (1 BED-NLR and 8 non-NLRs), and the BED domains of *YrNAM*, *Yr5*, *Yr7*, *YrSP*, *Xa1*, *Xo1* and *Rph15*. NLRs are shown in red and non-NLRs in black. All BED sequences are listed in Supplementary Data 8.

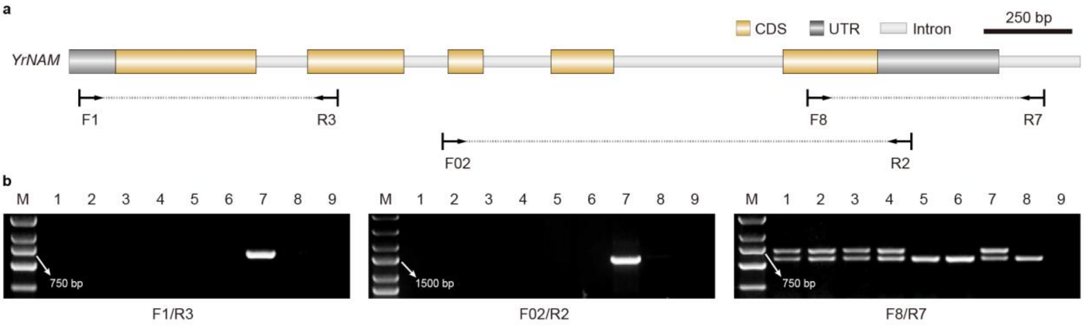

Supplementary Fig. 10. *YrNAM* distribution in *AF149112*-containing lines.

Three primer pairs, F1R3, F02R2, and F8R7, which amplify different regions of *YrNAM* (**a**) were used to determine the distribution of *YrNAM*. In a panel of 60 *AF149112*-containing lines[9-10], four lines were positive for F8R7, but none of them were positive for F1R3 and F02R2 (**b**). Lanes 1-9: Xi'anShixinmai, XiangNong 3, FengMai 2, FuFan 24, NanDa 2419, WangMai 17, P10-46, HXH, and H_2O (**b**). This experiment was performed independently two times with similar results. Source data are provided as a Source Data file.

Supplementary references

1. Alaux, M. *et al.* Linking the International Wheat Genome Sequencing Consortium bread wheat reference genome sequence to wheat genetic and phenomic data. *Genome Biol.* **19**, 111 (2018).
2. Avni, R. *et al.* Genome sequences of three *Aegilops* species of the section Sitopsis reveal phylogenetic relationships and provide resources for wheat improvement. *Plant J.* **110**, 179-192 (2022).
3. Huson, D. & Bryant, D. Application of phylogenetic networks in evolutionary studies. *Mol. Biol. Evol.* **23**, 254-67 (2006).
4. Katoh, K. & Standley D. M. MAFFT multiple sequence alignment software version 7: Improvements in performance and usability. *Mol. Biol. Evol.* **30**, 772 (2013).
5. Li, L. *et al.* Genome sequences of five Sitopsis species of *Aegilops* and the origin of polyploid wheat B subgenome. *Mol. Plant* **15**, 488-503 (2022)
6. Ma, S. *et al.* WheatOmics: A platform combining multiple omics data to accelerate functional genomics studies in wheat. *Mol. Plant* **14**, 1965-1968 (2021).
7. Marchal, C. *et al.* Comparative genomics and functional studies of wheat BED-NLR loci. *Genes* **11**: 1406 (2020).
8. Yuan, C. *et al.* Remapping of the stripe rust resistance gene *Yr10* in common wheat. *Theor. Appl. Genet.* **131**, 1253-1262 (2018).
