# Supplementary material for: Sequencing trait-associated mutations to clone wheat rust-resistance gene YrNAM
Source: Nat Commun. 2023 Jul 19;14:4353. doi: 10.1038/s41467-023-39993-2 (PMC10356923; doi:10.1038/s41467-023-39993-2)
Supplement: Supplementary file 3 — Description of Additional Supplementary Files [file 41467_2023_39993_MOESM3_ESM.pdf]

Description of Additional Supplementary Files

File Name: Supplementary Data 1
Description: Sequences of *YrNAM* homologs that were assembled from DNA resequencing of P10-46.

File Name: Supplementary Data 2
Description: Linkage analysis of *YrNAM* in F_2 population of M19/P10-46.

File Name: Supplementary Data 3
Description: Linkage analysis of *YrNAM* in $F_{2:3}$ population derived from Moro/HXH.

File Name: Supplementary Data 4
Description: Molecular marker analysis of recombinants between *Xpsp3000* and *Xsdauw79*.

File Name: Supplementary Data 5
Description: *Pst* infection tests of *YrNAM* native expression (PC1213) T_1 plants.

File Name: Supplementary Data 6
Description: *YrNAM* homologs annotated in common wheat and related species.

File Name: Supplementary Data 7
Description: Detection of *YrNAM* homologs in *Ae. longissima* and *Ae. sharonensis*.

File Name: Supplementary Data 8
Description: ZnF-BED domain sequences used in Supplementary Figure 9.

File Name: Supplementary Data 9
Description: Distribution of *YrNAM* in wheat cultivars positive for *AF149112*.

File Name: Supplementary Data 10
Description: Distribution of *YrNAM* in wheat cultivars and advanced lines.

File Name: Supplementary Data 11
Description: Primers used in this study.
