# Supplementary material for: Sequencing trait-associated mutations to clone wheat rust-resistance gene YrNAM
Source: Nat Commun. 2023 Jul 19;14:4353. doi: 10.1038/s41467-023-39993-2 (PMC10356923; doi:10.1038/s41467-023-39993-2)
Supplement: Supplementary file 15 — Reporting Summary [file 41467_2023_39993_MOESM15_ESM.pdf]

nature portfolio

Corresponding author(s): Jiajie Wu

Last updated by author(s): Jun 20, 2023

Reporting Summary

Nature Portfolio wishes to improve the reproducibility of the work that we publish. This form provides structure for consistency and transparency in reporting. For further information on Nature Portfolio policies, see our Editorial Policies and the Editorial Policy Checklist.

Statistics

For all statistical analyses, confirm that the following items are present in the figure legend, table legend, main text, or Methods section.

n/a	Confirmed	
☐	☒	The exact sample size (*n*) for each experimental group/condition, given as a discrete number and unit of measurement
☐	☒	A statement on whether measurements were taken from distinct samples or whether the same sample was measured repeatedly
☒	☐	The statistical test(s) used AND whether they are one- or two-sided<br>*Only common tests should be described solely by name; describe more complex techniques in the Methods section.*
☒	☐	A description of all covariates tested
☐	☒	A description of any assumptions or corrections, such as tests of normality and adjustment for multiple comparisons
☐	☒	A full description of the statistical parameters including central tendency (e.g. means) or other basic estimates (e.g. regression coefficient) AND variation (e.g. standard deviation) or associated estimates of uncertainty (e.g. confidence intervals)
☐	☒	For null hypothesis testing, the test statistic (e.g. *F*, *t*, *r*) with confidence intervals, effect sizes, degrees of freedom and *P* value noted<br>*Give P values as exact values whenever suitable.*
☒	☐	For Bayesian analysis, information on the choice of priors and Markov chain Monte Carlo settings
☒	☐	For hierarchical and complex designs, identification of the appropriate level for tests and full reporting of outcomes
☒	☐	Estimates of effect sizes (e.g. Cohen's *d*, Pearson's *r*), indicating how they were calculated

Our web collection on statistics for biologists contains articles on many of the points above.

Software and code

Policy information about availability of computer code

Data collection	The Isoform Sequencing (Iso-Seq) and whole-genome DNA resequencing data of wild type line 'P10-46' were generated from the PacBio Sequel II system and the Illumina NovaSeq 6000 platform, respectively. The RNA-Seq data of wheat cultivar 'Moro' and seven Pst-susceptible mutants were also generated from Illumina NovaSeq 6000 platform. The sequencing was performed by Berry Genomics (Beijing, China).<br>The ZnF-BED domain sequences used for Neighbour-network analysis were downloaded from Marchal et al., Genes 11, 1406 (2020).
Data analysis	1) STAM analysis<br>De novo transcriptome reconstruction: The PacBio subreads were processed by ccs (version 6.2.0) to generate polished circular consensus sequence (CCS reads), and processed by lima (version 2.2.0) for primer removal and demultiplexing. IsoSeq3 was used to trim PolyA tails and generate full-length, non-concatemer reads.<br>Construction of final transcriptome reference: Minimap2 (version 2.24) was used to align the full-length, non-concatemer reads to the genome reference, and RepeatMasker (version 4.1) was used to identify the transposable elements. Cupcake and Cogent (https://github.com/Magdoll) were used to generate unique transcripts for well-mapped reads and unmapped reads, respectively. The final transcript set was clustered using CD-HIT-EST (version 4.8.1).<br>Variant calling: Fastp (version 0.23.2) was used to generate clean RNA-Seq data of Pst-susceptible mutants and Moro. The high-quality sequencing reads were mapped to the reference using STAR (version 2.7.8a) and the mapped results were preprocessed using Picard (version 2.26). Variant calling was performed using GATK (version 4.2).<br>De novo assembly of DNA re-sequencing data: DNA re-sequencing data of P10-46 was mapped to the Poaceae repeat element database mipsREdat 9.3p (PGSB Repeat Database) using BWA (version 0.7.17) and a de novo assembly was performed using SPADES (version 3.15). A detailed description of STAM pipeline is available on GitHub (https://github.com/Feiny/STAM).<br>2) Phylogenetic tree analysis<br>Homologs/orthologs of YrNAM in common wheat and related species were acquired from WheatOmics (http://wheatomics.sdau.edu.cn/)

using BLAST (version 2.11.0), multiple sequence alignments were performed using MAFFT (version 7.475), the phylogenetic tree was built by the approximately maximum-likelihood method using FastTree (version 2.1.11). The neighbor-network was generated using SplitsTree4 (version 4.19).
3) Gene expression analysis
Using Barplot tool with default parameters in Hiplot Pro (https://hiplot.com.cn/).

For manuscripts utilizing custom algorithms or software that are central to the research but not yet described in published literature, software must be made available to editors and reviewers. We strongly encourage code deposition in a community repository (e.g. GitHub). See the Nature Portfolio guidelines for submitting code & software for further information.

Data

Policy information about availability of data

All manuscripts must include a data availability statement. This statement should provide the following information, where applicable:

- Accession codes, unique identifiers, or web links for publicly available datasets
- A description of any restrictions on data availability
- For clinical datasets or third party data, please ensure that the statement adheres to our policy

The YrNAM gene sequence has been deposited in NCBI Genbank under the accession numbers OP490604 (cDNA, T59230) and OP490605 (genomic DNA, NODE_33935). DNA resequencing data and error-corrected IsoSeq full-length cDNA reads for resistant WT P10-46, and RNA-Seq data for P10-46 and seven independent mutants have been deposited in the NCBI Sequence Read Archive (SRA) under accession number PRJNA877303.
The IWGSC RefSeq v2.1 were downloaded from https://wheat-urgi.versailles.inra.fr/Seq-Repository; RNA-Seq expression data of Chinese Spring were downloaded from ENA database under accession number ERP004714; the PGSB database mipsREdat 9.3p was downloaded from http://pgsb.helmholtz-muenchen.de/plant/index.jsp.

Research involving human participants, their data, or biological material

Policy information about studies with human participants or human data. See also policy information about sex, gender (identity/presentation), and sexual orientation and race, ethnicity and racism.

Reporting on sex and gender	Not applicable
Reporting on race, ethnicity, or other socially relevant groupings	Not applicable
Population characteristics	Not applicable
Recruitment	Not applicable
Ethics oversight	Not applicable

Note that full information on the approval of the study protocol must also be provided in the manuscript.

Field-specific reporting

Please select the one below that is the best fit for your research. If you are not sure, read the appropriate sections before making your selection.

☒ Life sciences ☐ Behavioural & social sciences ☐ Ecological, evolutionary & environmental sciences

For a reference copy of the document with all sections, see nature.com/documents/nr-reporting-summary-flat.pdf

Life sciences study design

All studies must disclose on these points even when the disclosure is negative.

Sample size	The sample size of STAM analysis was determined according to previous reports in identifications of a gene using EMS mutants, Steuernagel et al., Nat Biotechnol. 2016, 34(6):652-655, Sánchez-Martín et al., Genome Biology 2016, 17:221.
Data exclusions	No data were excluded.
Replication	Molecular experiments including PCR tests for transgenic plants, gene expression, gene distribution, and so on, are repeated at least twice independently. The number of independent trials is now stated in the Fig. legends.
Randomization	To perform Pst tests for transgenic plants expressing YrNAM, around 20 seeds were randomly chosen from each T1 line. After phenotyping, T2 seeds were harvested from randomly 3-5 plants from each line. In further tests, around 20 seeds were chosen from one random T2 plant from each line. To investigate the distribution of YrNAM in wheat germplasms including cultivars, advanced breeding lines and relatives of wild wheat, samples were not randomly chosen but were all what we have conserved in the lab.
Blinding	In the experiments of phenotyping, the investigators collected the infection type (ITs) of each transgenic plant before checking whether or not

Blinding	it is transgenic positive.

Reporting for specific materials, systems and methods

We require information from authors about some types of materials, experimental systems and methods used in many studies. Here, indicate whether each material, system or method listed is relevant to your study. If you are not sure if a list item applies to your research, read the appropriate section before selecting a response.

Materials & experimental systems

n/a	Involved in the study
☒	☐ Antibodies
☒	☐ Eukaryotic cell lines
☒	☐ Palaeontology and archaeology
☒	☐ Animals and other organisms
☒	☐ Clinical data
☒	☐ Dual use research of concern
☒	☐ Plants

Methods

n/a	Involved in the study
☒	☐ ChIP-seq
☒	☐ Flow cytometry
☒	☐ MRI-based neuroimaging
